# The Muscle Fiber Profiles, Mitochondrial Content, and Enzyme Activities of the Exceptionally Well-Trained Arm and Leg Muscles of Elite Cross-Country Skiers

**DOI:** 10.3389/fphys.2018.01031

**Published:** 2018-08-02

**Authors:** Niels Ørtenblad, Joachim Nielsen, Robert Boushel, Karin Söderlund, Bengt Saltin, Hans-Christer Holmberg

**Affiliations:** ^1^Department of Sports Science and Clinical Biomechanics, SDU Muscle Research Cluster, University of Southern Denmark, Odense, Denmark; ^2^School of Kinesiology, University of British Columbia, Vancouver, BC, Canada; ^3^Åstrand Laboratory, The Swedish School of Sport and Health Sciences, Stockholm, Sweden; ^4^Copenhagen Muscle Research Centre, Copenhagen, Denmark; ^5^Swedish Winter Sports Research Centre, Mid Sweden University, Östersund, Sweden; ^6^School of Sport Sciences, UiT The Arctic University of Norway, Tromsø, Norway

**Keywords:** limb muscles, fiber plasticity, training, capillarization, mitochondria, IMCL, cross-country skiing

## Abstract

As one of the most physically demanding sports in the Olympic Games, cross-country skiing poses considerable challenges with respect to both force generation and endurance during the combined upper- and lower-body effort of varying intensity and duration. The isoforms of myosin in skeletal muscle have long been considered not only to define the contractile properties, but also to determine metabolic capacities. The current investigation was designed to explore the relationship between these isoforms and metabolic profiles in the arms (*triceps brachii*) and legs (*vastus lateralis*) as well as the range of training responses in the muscle fibers of elite cross-country skiers with equally and exceptionally well-trained upper and lower bodies. The proportion of myosin heavy chain (MHC)-1 was higher in the leg (58 ± 2% [34–69%]) than arm (40 ± 3% [24–57%]), although the mitochondrial volume percentages [8.6 ± 1.6 (leg) and 9.0 ± 2.0 (arm)], and average number of capillaries per fiber [5.8 ± 0.8 (leg) and 6.3 ± 0.3 (arm)] were the same. In these comparable highly trained leg and arm muscles, the maximal citrate synthase (CS) activity was the same. Still, 3-hydroxy-acyl-CoA-dehydrogenase (HAD) capacity was 52% higher (*P* < 0.05) in the leg compared to arm muscles, suggesting a relatively higher capacity for lipid oxidation in leg muscle, which cannot be explained by the different fiber type distributions. For both limbs combined, HAD activity was correlated with the content of MHC-1 (*r*^2^ = 0.32, *P* = 0.011), whereas CS activity was not. Thus, in these highly trained cross-country skiers capillarization of and mitochondrial volume in type 2 fiber can be at least as high as in type 1 fibers, indicating a divergence between fiber type pattern and aerobic metabolic capacity. The considerable variability in oxidative metabolism with similar MHC profiles provides a new perspective on exercise training. Furthermore, the clear differences between equally well-trained arm and leg muscles regarding HAD activity cannot be explained by training status or MHC distribution, thereby indicating an intrinsic metabolic difference between the upper and lower body. Moreover, trained type 1 and type 2A muscle fibers exhibited similar aerobic capacity regardless of whether they were located in an arm or leg muscle.

## Introduction

Among the most demanding of Olympic sports, cross-country skiing competitions on varying terrain require the use of a variety of skiing techniques that involve the upper and/or lower body to different extents. In recent decades, this primarily endurance sport has changed to include novel events such as pursuit, mass-start, and sprint races, with head-to-head competitions and a wider range of speeds. Improved track preparation, equipment, and skiing techniques, in combination with more effective training (especially of upper-body strength/power and endurance), have elevated racing speeds in general (Holmberg, 2015).

The necessity for today’s elite cross-country skier to combine considerable endurance with rapid generation of high forces during short contacts with the ground has enhanced focus on optimizing related morphological and metabolic adaptations in the skeletal muscles of the upper and lower body (Holmberg et al., 2005). The relatively unique situation that both the leg and arm muscles of elite cross-country skiers are highly trained has allowed important comparisons that have helped provide novel insights into the limits of physiological regulation and performance, thereby helping to improve training routines.

Skeletal muscles are composed of motor units, containing muscle fibers with the same specific characteristics (Canepari et al., 2010). In general, muscle fibers are distinguished from one another on the basis of (1) the contractile apparatus [myosin heavy chain (MHC) or ATPase isoforms]; (2) contractile characteristics (fast vs. slow twitch); (3) Ca^2+^ handling properties and metabolic profile (oxidative or glycolytic), with the golden standard being the MHC-isoform (Schiaffino and Reggiani, 2011). The functional significance of the MHC isoform for its contractile characteristics is well established (Schiaffino and Reggiani, 2011), even for hybrid fibers co-expressing MHC isoforms. The metabolic capacity of the muscle fiber is dependent on the degree of capillarization, substrate availability and mitochondrial content, while the Ca^2+^ handling properties are dependent on sarcoplasmic reticulum (SR) content and property (Stephenson et al., 1998; Ørtenblad et al., 2000b; Gejl et al., 2014). The metabolic and Ca^2+^ handling properties are generally considered as being linked with contractile fiber type characteristics. Human muscle fibers expressing MHC-1 have the highest oxidative capacity while having slow shortening velocity (incl. excitation–contraction coupling) and slower Ca^2+^ handling, whereas MHC-2 fibers have the opposite characteristics. However, metabolic variation within each fiber type and fibers in arm and leg muscle is less well explored, both with regard to extent and influence on the metabolic response of the fiber.

Most Olympic disciplines involve mainly the legs and lower body, with fewer combining upper and lower body as in cross-country skiing. Despite the importance of the arms in sports such as swimming, rowing, and cross-country skiing, our knowledge of arm muscle physiology is considerably less than in the case of the legs and warrants more attention. The few direct comparisons of arm and leg muscles indicate that arm muscles are less oxidative and less capable of extracting oxygen from the circulation, irrespective of training status, with greater variability in blood flow during exercise (Van Hall et al., 2003; Calbet et al., 2005). Furthermore, exercising arm muscle has evidently a lower fat oxidation compared to leg muscle (Calbet et al., 2005; Helge, 2010). However, the physiological comparison of arms and legs is hampered by an often-unequal training status of the limbs. Thus, direct comparisons of the highly trained arm and leg muscles of elite cross-country skiers can be made unequivocally.

Accordingly, the current investigation assessed further the metabolic capacity in the upper and lower body of such skiers, as well the potential relationship between the various isoforms of MHCs and metabolic profile. For this purpose, we examined type 1 and type 2 fibers from leg (*vastus lateralis*) muscle and arm muscle (*triceps brachii*) from successful cross-country skiers with exceptionally well-trained lower and upper body. Our hypotheses were that (1) there are intrinsic metabolic differences between equally well-trained arm and leg muscles and (2) type1 and type 2 muscle fibers possess similarly metabolic capacity, regardless of their location in an arm or leg muscle and that this possible adaptation is not linked to the isoform of the muscle fibers.

## Materials and Methods

### Subjects

Ten elite male Norwegian cross-country skiers participated in the study, as part of a larger project and related data from the project has already been published (Nielsen et al., 2011; Ørtenblad et al., 2011; Koh et al., 2017). Their mean (±SD) age, height, weight, and VO_2max_ were 22 ± 1 yr, 181 ± 2 cm, 79 ± 8 kg, and 5.37 ± 0.46 L⋅min^-1^ (69 ± 5 ml⋅kg^-1^⋅min^-1^), respectively (**Table 1**) and a hematocrit of 47 ± 1% and hemoglobin of 155 ± 2 mmol/l. These skiers had trained systematically for an average of 11 years; six had competed as members of the Norwegian national team; and eight competed in the FIS World Cup the year after this study, with one winning a World Cup race (**Table 1**). All subjects were informed of the test procedures and potential risks prior to providing their written informed consent to participate. The research procedures and experimental protocol were pre-approved by the Human Ethics Committee of Umeå University, Sweden (#07-076M), and performed in accordance with the Declaration of Helsinki.

**Table 1:** Characteristics of the 10 elite male cross-country skiers who participated in this study.

Subject	Age (years)	Weight (kg)	Height (cm)	VO_2_ max (L⋅min^-1^)	VO_2_ max (mL⋅kg^-1^⋅min^-1^)	Performance
1	22	81.4	190	5.82	71.5	12^th^ in WC 50-km C (2012)
2	21	77.2	182	5.10	66.1	among the top 30/15 in NOR Tr and Sp, respectively
3	22	87.3	188	6.08	69.6	among the top 30 in NOR Tr
4	19	76.0	178	5.21	68.6	12^th^ in NNC Sp (2009)
5	21	77.2	178	5.16	66.8	40^th^ in NNC 15F (2011)
6	23	66.8	172	5.30	79.3	9^th^ in WC 15-km F (2008)
7	23	92.4	193	6.05	65.5	14^th^ in WC Sp (2011)
8	23	87.1	179	5.34	61.3	Among the top 50 and 30 in NOR Tr and Sp, respectively
9	24	69.9	175	4.82	69.0	Among the top 30 in NOR Sp
10	22	72.5	173	4.85	66.9	Among the top 60 in NOR
Mean ± SD	22 ± 1	78.8 ± 8.2	181 ± 7	5.37 ± 0.46	68.5 ± 4.7	


*WC, World Cup; NC, Norwegian National Championship; Tr, traditional/longer distances; Sp, sprint distances; C, classical technique; F, free technique.*

### Procedures

#### Laboratory Tests

VO_2max_ was determined during diagonal skiing with roller skis on a treadmill (Rodby, Södertälje, Sweden; Calbet et al., 2005), starting at 11 km⋅h^-1^ on a treadmill inclination of 4° and increasing the incline by 1° each minute until exhaustion. During the tests, each subject was secured with a safety harness suspended from the ceiling. For the subjects, roller skiing on the treadmill was a regular part of their training.

Respiratory variables were determined with the mixed expired gas procedure, employing an ergo-spirometry system (AMIS 2001 model C, Innovision A/S, Glamsbjerg, Denmark) equipped with an inspiratory flowmeter. The gas analyzers were calibrated with a high-precision mixture of 16.0% O_2_ and 4.0% CO_2_ (Air Liquide, Kungsängen, Sweden) and the flowmeter calibrated at low, medium, and high flow rates with a 3-l air syringe (Hans Rudolph, Kansas City, MO, United States). Ambient conditions were monitored with an external apparatus (Vaisala PTU 200, Vaisala OY, Helsinki, Finland). Expired O_2_ and CO_2_ and the inspired minute ventilation (V Ė) were monitored continuously and VO_2_ values averaged during the final 30 s at each workload. Heart rate was recorded continuously by the Polar S610 monitor (Polar Electro Oy, Kempele, Finland).

#### Muscle Biopsy Preparation and Analysis

Muscle biopsies were taken from leg and arm muscles and standardization of the location on the muscle and muscle depth was ensured. After local anesthesia (2–3 ml 2% lidocaine), an incision was made through the skin and fascia and the muscle biopsy was taken from the *vastus lateralis* (leg) and *triceps brachii* (distal part of the lateral head, arm), using a modified Bergström needle with suction. These muscles were selected because they are very active during cross-country skiing (Komi and Norman, 1987; Holmberg et al., 2005). The skiers had four biopsies taken from both arm and leg muscle. The muscle specimen was dried on filter paper and placed on a glass plate cooled on ice. After the removal of visible connective tissue and fat, each muscle specimen was divided into four specimens then handled in the following ways: (1) frozen directly in liquid N_2_ and stored for later analyses of enzyme activity and glycogen content; (2) fixed for transmission electron microscopy (TEM) analysis; (3) 10–20 mg was mounted in an embedding medium (OCT compound), frozen rapidly in isopentane pre-cooled with liquid N_2_, and stored at -80°C for later histochemical analysis; or (4) a segment was weighed and homogenized in 10 volumes (wt/vol) of ice-cold buffer (300 mM sucrose, 1 mM EDTA, 10 mM NaN_3_, 40 mM Tris-base, and 40 mM histidine at pH 7.8) at 0°C in a 1-ml glass homogenizer with a glass pestle (Kontes Glass Industry, Vineland, NJ, United States). Prior to homogenization, the muscle sample was rinsed free of contaminating blood by washing it in an ice-cold buffer. The homogenate was analyzed for protein content and MHC composition. All in all 40 biopsies were obtained from the leg and arm muscles, and in one biopsy from arm, the sample portion was not large enough to obtain CS activity.

#### Myosin Heavy Chain Composition

Myosin heavy chain composition was analyzed using gel electrophoresis. Briefly, muscle homogenate (80 μl) was mixed with 200 μl sample buffer (10% glycerol, 5% 2-mercaptoethanol, 2.3% SDS, 62.5 mM Tris-base, and 0.2% bromophenolblue at pH 6.8), boiled in a water bath at 100°C for 3 min, and loaded with three different amounts of protein (10–40 μl) on an SDS-PAGE gel [6% polyacrylamide (100:1, acrylamide:bis-acrylamide), 30% glycerol, 67.5 mM Tris-base, 0.4% SDS, and 0.1 mM glycine]. Gels were run at 80 V for at least 42 h at 4°C and MHC bands made visible by staining with Coomassie and three separate bands could be detected and characterized as MHC-1, MHC-2A, and MHC-2X. The gels were scanned (Linoscan 1400 scanner, Heidelberg, Germany) and the MHC bands were quantified densitometrically (Phoretix 1D, nonlinear, Newcastle, United Kingdom). MHC-2 was identified with Western blot using monoclonal antibody (Sigma M 4276) with the Xcell IITM protocol (Invitrogen, Carlsbad, CA, United States). All values presented are the means of three biopsies (two from one leg/arm and one from the other leg/arm), utilizing three different concentrations of protein from each biopsy.

#### Enzyme Activity

The maximal activities of 3-hydroxy-acyl-CoA-dehydrogenase (HAD) and citrate synthase (CS), were determined fluorometrically at 25°C (Lowry and Passonneau, 1972) in freeze-dried muscle dissected free of non-muscle constituents. CS activity was determined by the addition of oxaloacetate to a buffer solution containing muscle homogenate, DTNB buffer, acetyl-CoA. HAD activity was measured after the addition of acetoacetyl-CoA to a buffer solution containing imidazole, NADH and EDTA. Absorbance of CS and HAD was recorded for 600 s, converted into enzyme activity rates, and expressed as μmol⋅g^-1^ dw⋅min^-1^.

#### Histochemical Analysis of Capillarization and ATPase Fiber Typing

Histochemical analysis of ATPase (Brooke and Kaiser, 1970) was used to determine the fiber type composition (type 1, 2a, 2x) and fiber cross-sectional area (CSA), while the amylase periodic acid-Schiff reaction (Andersen, 1975) was applied for staining of capillaries (TEMA image analysis system; Scanbeam a/s, Hadsund, Denmark). In brief, serial sections (10 μm) of the muscle biopsies samples were cut in a cryostat at -20°C, and fiber type distribution was obtained by ATPase histochemistry analysis performed after pre-incubation at pH 4.37, 4.60, and 10.30. An average of 85 ± 16 fibers was analyzed in each biopsy. The serial sections of the various ATPases were visualized and analyzed for fiber type, using a TEMA image analyzing system (Scanbeam, Hadsund, Denmark).

#### Transmission Electron Microscopy

**FIGURE 1:**
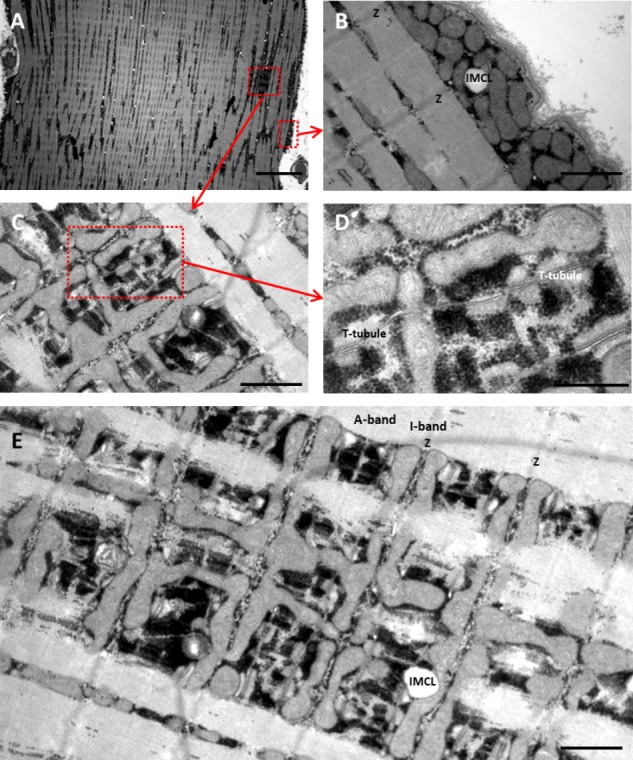
TEM images showing the subcellular localization of skeletal muscle mitochondria. All images are from leg muscle (*vastus lateralis*). **(A)** Overview of a part of fiber showing the myofibrillar (Myo) space and subsarcolemmal (SS) space. **(B)** The typical localization of SS mitochondria (mit) in skeletal muscle, also showing a intramyocellular lipid (IMCL). **(C)** In the Myo space, intermyofibrillar mitochondria are wrapped around the myofibrils, mainly in the I-band and often connected to an adjacent mitochondrion through the A-band. There is less marked connection between neighboring mitochondria in the I-band. **(D)** Intermyofibrillar mitochondria in the I-band on each side of the Z-line, with the t-tubular system (t-system) and mitochondria intertwined. **(E)** Overview demonstrating the IMF mitochondria are mainly located in the I-band on each side of the z-line and often connected to an adjacent mitochondrion in the same sarcomere through the A-band. All the gray structures in the fiber are mitochondria with slightly visible inner cristae. Glycogen granules can be seen as black dots. Z, Z-line; A, A-band; IMCL, intramyocellular lipid; T-tubule, transverse tubular system. Scale bar: **A**, 10 μm; **B**,**C**, 1 μm; **D**, 0.5 μm; **E**, 1 μm. Original magnification: **A**, x1,600; **B,C**, x20,000; **D**, x50,000; **E**, x13,000.

To examine the content and subcellular localization of mitochondria and lipids, muscle biopsy specimens were prepared for TEM as described previously (Nielsen et al., 2010a,b). In the prepared sections, all longitudinal-oriented fibers (∼9 per biopsy) were photographed at x40,000 magnification in a randomized, systematic order to ensure unbiased results. From each fiber, 12 images both from the myofibrillar (six from the superficial and central region, respectively) and subsarcolemmal (SS) regions were obtained as previously described (Nielsen et al., 2010a,b). Fibers were identified as type 1 or type 2 based on a combination of mitochondrial volume fraction and z-line width as described elsewhere (Nielsen et al., 2011). In order to identify the two main fiber types, all intermediate fibers were discarded and only distinct type 1 and 2 fibers were included, respectively (*n* = 2–3 fibers of each type per biopsy). The contents of mitochondria in the intermyofibrillar (IMF) and SS regions were estimated by point counting (Weibel, 1980, **Figure 1**). IMF mitochondria is expressed as volume fractions of the myofibrillar space and the values for the superficial region were weighted three times higher than those for the central region, to account for the cylindrical shape of the fibers, in which the superficial region (outermost half of the diameter) occupies three-quarters of the volume. The SS mitochondria are expressed as volume per surface area of the muscle fiber. The estimated coefficient of error (_est_CE; see Howard and Reed, 2005) was 0.18 and 0.24 for IMF and SS mitochondria, respectively, with no difference between legs and arms. Total volume fractions of mitochondria and lipids, respectively (IMF + SS), were obtained by recalculating the SS subfractions relative to myofibrillar volume density, assuming a cylindrical shape of the fibers and a radius of 40 μm, as previously described (Nielsen et al., 2010a).

### Statistical Analyses

All values presented are means ± standard error of the mean (SEM) and were subjected to ANOVA test, with significant differences between means identified using the Bonferroni *post hoc* test (GraphPad Prism 6.07). All interactions or main effects were examined using a linear mixed-effects model, with the subject, limb, fiber type, and fiber as random effects and limb, fiber type, and location as fixed effects, using the Stata 10.1 software (StataCorp. 2007; Stata Statistical Software: Release 10; StataCorp LP, College Station, TX, United States). Variables exhibiting skewed distributions were log-transformed prior to analysis. The level of significance was set at α = 0.05.

## Results

### Fiber Type Distribution

The MHC distribution in the *vastus lateralis* and *triceps brachii* was the same on the left- and right-hand sides, with a significantly higher proportion of MHC-1 in the legs (58 ± 2%, range [34–69%]) than the arms (40 ± 3%, range [24–57%]) (*P* < 0.01, **Table 2**). Accordingly, the proportion of MHC-2A in the legs was lower (41 ± 2 vs. 60 ± 3%). The average MHC distribution showed a considerable variation between the skiers with MHC-1 ranging between 34–69% (leg) and 24–57% (arm) (**Table 2**). Notably, the two skiers with the highest proportion of MHC-2A in arms (70 and 72%) were successful sprint skiers.

**Table 2:** The profile of myosin heavy chains and enzyme activities in the arm (*triceps brachii*) and leg (*vastus lateralis*) muscles of elite cross-country skiers (*n* = 10).

	Fiber type distribution (% of total)			Enzyme activity		
		
	MHC-1	MHC-2A	MHC-2X	CS	HAD	HAD/CS
Leg	58 ± 2	41 ± 2	1.0 ± 0.4	118 ± 6	144 ± 12	1.22
Arm	40 ± 3^∗^	60 ± 3^∗^	0.4 ± 0.2	111 ± 10	95 ± 12^∗^	0.84^∗^


*The maximal activities of 3-hydroxy-acyl-CoA-dehydrogenase (HAD) and citrate synthase (CS) are given in μmol/g dw/min. ^∗^Significantly different from the leg muscle.*

### Enzyme Activities

**FIGURE 2:**
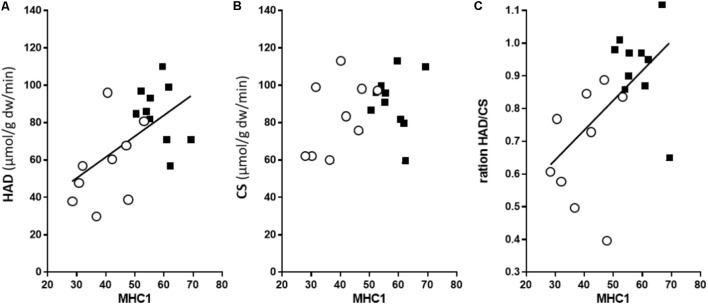
The relationships between the percentage of MHC-1 and 3-hydroxyacyl-CoA dehydrogenase activity (HAD, *n* = 10) **(A)**; citrate synthase activity (CS, *n* = 9) **(B)**; and relative capacity to oxidize fat (HAD/CS ratio) **(C)**. The open circles depict data for the arms and the leg black squares for the legs. For the arm and leg combined, there was a significant correlation between the MHC-1 content and HAD activity (*r*^2^ = 0.32, *P* = 0.011), as well as the HAD/CS ratio (*r*^2^ = 0.27, *P* = 0.021).

The maximal CS activity of well-trained arm and leg muscles was the same (**Table 2**), despite the higher MHC-1 content of the legs, thus demonstrating a non-MHC-dependency in the CS activity. In contrast, the maximal activity of the key enzyme in the ß-oxidation, HAD, was 52% higher (*P* < 0.05) in the leg compared to arm muscles. Accordingly, the ratio between the HAD and CS activity was 45% higher in leg than arm (1.22 in the leg and 0.86 in arm, *P* < 0.01), suggesting a relatively higher capacity for lipid oxidation in leg muscle. Further, there was no association between CS activity and MHC distribution (**Figure 2A**). Thus, CS activity in these highly trained muscles is not associated with the MHC distribution. In contrast, MHC-1 content was a robust predictor of HAD capacity (*P* = 0.011, *r*^2^ = 0.32, **Figure 2B**). In line with this, there was also a strong correlation between HAD/CS ratio and the MHC-1 content (*P* = 0.021, *r*^2^ = 0.27), with no association in trained leg (**Figure 2C**). Taken together, in these highly trained skiers, there is a close association between MHC distribution and both absolute (HAD) and relative (HAD/CS) capacity to oxidize fat, with no association between CS capacity and MHC distribution.

### Fiber Capillarization and Size

The total number of capillaries per total number of fibers and the number of capillaries per fiber area were not different between leg and arm muscle, averaging 2.9 ± 0.1 capillaries per fiber and 417 ± 14 capillaries/mm^2^ (**Table 3**). The average number of capillaries around each fiber was 5.8 ± 0.8 for the leg and 6.3 ± 0.3 for the arm. When considering capillaries around each fiber type, there were significantly fewer capillaries in type 2x fibers as compared with type 1 and 2a in leg muscle (*P* < 0.05, **Table 3**). There were no fiber type differences in arm muscle; however, there was a tendency toward a higher capillarization in type 2a fibers (*P* = 0.078). Further, there was a clear difference in capillarization between leg and arm muscle in type 2a fibers, with 14% more capillaries per fiber in arm muscle (**Table 3**).

**Table 3:** Capillary density in the arm (*triceps brachii*) and leg (*vastus lateralis*) muscles of elite cross-country skiers (*n* = 10).

	#cap/fiber	cap/mm^2^	Type 1	Type 2a	Type 2x	Average
Leg	2.8 ± 0.1	437 ± 22	5.9 ± 0.3	5.9 ± 0.2	5.1 ± 0.3^#^	5.8 ± 0.8
Arm	3.0 ± 0.2	394 ± 14	5.8 ± 0.3	6.7 ± 0.3^∗^	6.0 ± 2.0^∗^	6.3 ± 0.3


*Capillary density was assessed immunohistochemically. Number of capillaries is given in: total number of capillaries per total number of fibers (#cap/fiber); total number of capillaries per muscle area (cap/mm^2^), and number of capillaries around each fiber for each fiber type and average for all fibers. ^∗^Significantly different from the corresponding value for leg muscle; ^#^significantly different from the corresponding values for the other fiber types.*

The average fiber size for each fiber type and hybrid fibers, in arm and leg muscles, is shown in **Table 4**. There was no significant difference in mean fiber size between fiber types in leg muscle. However, in arm muscle, type 2a fibers were significantly larger than type 1 fibers (*P* < 0.05).

**Table 4:** Fiber size in the arm (*triceps brachii*) and leg (*vastus lateralis*) muscles of elite cross-country skiers (*n* = 10).

	Type 1	Type 2a	Type 2x	Type 2a/x
Leg	5423 ± 272	6811 ± 297	6590 ± 363	5840 ± 518
Arm	5356 ± 200^∗¥¤^	8105 ± 394^∗^	6125 ± 960^¥^	4576 ± 176^¥^


*Fiber size (in μm^2^) was assessed immunohistochemically. ^∗^Significantly different from the corresponding value for leg muscle; ^¥^ significantly different from type 2a fibers; ^¤^significantly different from type 2x fiber.*

Estimation of the number of capillaries per individual fiber area in trained muscles demonstrated that type 1 fibers in both leg and arm muscles had, on average, 27% higher capillarization than type 2 fibers (*P* < 0.05), with no difference between limbs. Thus, a higher number of capillaries per fiber type 2a fibers of the arm are linked with a larger fiber size.

### Mitochondrial Content and Subcellular Localization

**FIGURE 3:**
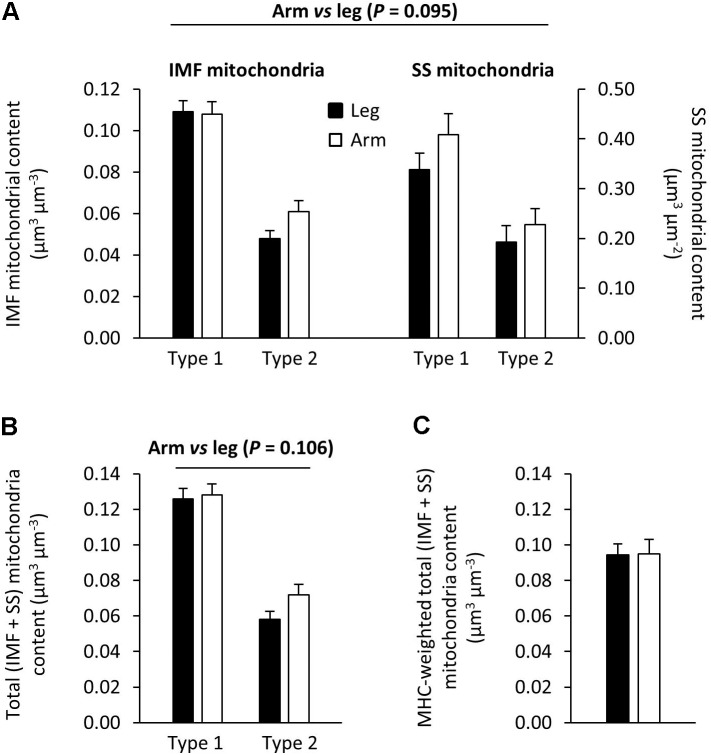
Mitochondria content and subcellular localization in distinct fiber types and at whole-muscle level of leg and arm muscles. There was a tendency (*P* = 0.095) toward a higher mitochondrial content in the intermyofibrillar (IMF) and subsarcolemmal (SS) regions, of arm muscle (open bars) compared with leg muscle (filled bars) **(A)**. This tendency is also apparent when calculating total mitochondrial content (IMF + SS) **(B)**. **(C)** Weighted mitochondrial volumes in the arm and leg muscle, estimated from a fiber type distribution of 57 and 37% MHC-I for the leg and arm (*n* = 9), respectively. These MHC weighted values of whole-muscle mitochondrial content in arm and leg muscles are similar. Values are means ± SE (*n* = 29–30 fibers from 10 subjects).

Transmission electron microscopy images showing the subcellular localization of skeletal muscle mitochondria in the highly trained cross-country skiers are shown in **Figure 1**, clearly demonstrating a very high mitochondrial volume in these trained muscles. The SS mitochondria were unevenly distributed below the sarcolemma, with a higher volume located near the capillaries and around the nuclei. The IMF mitochondria are wrapped around the myofibrils, mainly located on each side of the z-line. These mitochondria in the I-band are often connected to an adjacent mitochondrion in the same sarcomere through the A-band. Individual values for the total volume of mitochondria per volume of myofiber are given in **Table 5**. The total volume of mitochondria is a volume-weighted average of the superficial region and the central region of the myofiber as well as the SS space. The individual values are based on 8–12 myofibers from two different biopsies. The total mitochondrial volume averaged 8.6 ± 1.6 and 9.0 ± 2.0 μm^3^⋅μm^-3^, for the arm and leg, respectively. The relative distribution of the mitochondrial subcellular regions was estimated in a total of 29 or 30 fibers from the 10 participants. In these highly endurance-trained athletes, the skeletal muscle mitochondria had similar relative distribution between IMF and SS localizations in both leg and arm muscles and in type 1 and 2 fibers. Thus, 83–86% of the mitochondria are localized in the IMF region and 11–14% in the SS region. The mitochondrial content and subcellular localization in distinct fiber types and at the whole-muscle level of leg and arm muscles is shown in **Figure 3**. Intriguingly, there was a tendency toward (10–20%) a lower mitochondrial content in the IMF and SS regions of leg muscle fibers compared with arm muscle fibers (**Figure 3A**, *P* = 0.095). This is also apparent when calculating a total (IMF + SS) mitochondrial content (**Figure 3B**). By taking the different MHC composition of leg and arm muscles into account, the average fiber type-mitochondrial volume can be estimated, given a fiber type distribution of 57 and 37% MHC-1 in leg and arm, respectively. Weighting the fiber type distribution, the whole-muscle mitochondrial volume in leg and arm muscle was similar (**Figure 3C**). Thus, at the whole-muscle level, the non-significantly higher mitochondrial content in the arms mediated, despite a relatively higher number of MHC-2 fibers, an equal whole-muscle mitochondrial content in the legs and arms (**Figure 3C**). There was a significant correlation (*P* = 0.02) between the total mitochondrial content in arm muscle and whole body VO_2 max_ (L⋅min^-1^), which was not apparent in leg muscle.

**Table 5:** The volume of mitochondria – total and in the superficial and central intermyofibrillar space (IMF) and the sarcolemmal space (SS) – in the arm and leg muscles of elite cross-country skiers (*n* = 10).

Participant	Arm (*triceps brachii*)				Leg (*vastus lateralis*)			
		
	Total	IMF_Superficial_	IMF_Central_	SS	Total	IMF_Superficial_	IMF_Central_	SS
1	9.7	10.2	4.1	0.21	–	–	–	–
2	10.1	9.8	5.2	0.28	7.1	6.6	4.4	0.20
3	12.7	11.5	5.8	0.53	9.4	9.2	4.3	0.29
4	9.4	9.0	2.9	0.38	9.1	7.9	4.6	0.41
5	8.1	6.7	5.3	0.34	8.1	7.9	4.4	0.23
6	5.6	5.8	2.4	0.13	6.2	5.7	3.6	0.21
7	7.4	6.8	5.5	0.19	9.3	9.2	4.7	0.25
8	10.6	10.1	4.7	0.36	11.8	11.0	7.0	0.37
9	8.6	7.6	5.9	0.29	8.8	8.9	3.9	0.24
10	8.0	7.7	2.4	0.33	7.6	6.9	4.5	0.25
Mean	9.0	8.5	4.4	0.31	8.6	8.1	4.6	0.27
SD	2.0	1.9	1.4	0.11	1.6	1.6	1.0	0.07


*Total, total volume of mitochondria per volume of myofiber; IMF_*superficial*_, intermyofibrillar mitochondrial volume per volume of myofibrillar space in the superficial region of the myofiber; IMF_*central*_, intermyofibrillar mitochondrial volume per volume of myofibrillar space in the central region of the myofiber; SS, subsarcolemmal mitochondrial volume per area of myofiber surface. The individual values presented are the means for 8–12 myofibers in biopsies taken before and 22 h after the race. All data are given in volume densities (μm^*3*^ μm^-*3*^), except in the case of SS, where they are volume per SS area (μm^*3*^⋅μm^-*2*^).*

## Discussion

Here we compare equally trained limb muscles from elite cross-country skiers. A key finding here was that the mitochondrial volume percentage and CS activity is equal in legs and arms, despite the presence of a higher proportion of MHC-2 fibers in the arms. Furthermore, we demonstrate that well-trained type 1 and type 2 muscle fibers can have similar capillarization, regardless of whether they are located in arm or leg muscle and that the capillarization is not linked with the muscle fiber type, indicating a divergence between fiber type pattern and aerobic metabolic capacity. Also, comparable highly trained leg and arm muscles exhibited clear difference in their enzyme-linked ability to oxidize fatty acids (HAD capacity) and combined with previous data on a fourfold higher intramyocellular lipid (IMCL) volume contents in leg muscles; this points to a clear limb difference in fat metabolism between the leg and the arm, which cannot be explained by the different fiber type distributions.

### Fiber Type Malleability

In order to fulfill various functional needs, different skeletal muscle fiber types express different molecular isoforms of myosin. The contractile characteristics of the given muscle fiber type are generally considered as being linked with metabolic and Ca^2+^ handling properties, with fibers expressing MHC-1 having the highest oxidative capacity while being slow to shorten and having slower Ca^2+^ handling, with MHC-2 fibers having the opposite characteristics. This was demonstrated very clearly in the early studies by Burke et al. (1971), who showed a phenotypic characterization of quite strict links between contractile function and metabolic profile in that type 2 fibers are glycolytic, while type 1 fibers are oxidative. Despite several reports indicative of plasticity in this relationship, this long-held concept is still the reigning dogma. In later studies on humans, more evidence has been provided on the large plasticity of all fiber types with respect to their aerobic potential despite no or only a small transformation of the type 2a to the type 1 isoform (Holloszy, 1967; Hoppeler and Fluck, 2003; Schiaffino and Reggiani, 2011). In line with this, Essén et al. (1975) reported an equally high SDH activity in the type 2 and type 1 muscle fibers in top endurance runners [with a maximal oxygen uptake (VO_2max_) > 72 ml⋅kg^-1^⋅min^-1^], with untrained having a clear fiber type difference with only half the SDH activity in their type 2 muscle fibers. Also, the mitochondrial volume density is generally considered to be strongly fiber type-dependent. In untrained humans, the mitochondrial volume varies from 6% in type I fibers to 4.5% in type 2a and 2.3% in type 2x fibers (Howald et al., 1985), with a more pronounced difference in animal studies of oxidative and glycolytic muscle, i.e., 2.7 times higher in rabbits and 4.5 times higher in rats (Saltin and Gollnick, 1983; Jackman and Willis, 1996). In the current study, we compared equally trained arm and leg muscle based on the same CS activity (**Table 2**), the same average capillarization (**Table 3**), and no difference in the mitochondrial content at whole-muscle level. Based on this, we state that arm and leg muscle are equally trained. In these endurance-trained humans, there is a twofold higher mitochondrial volume density between type 1 and 2 fibers (**Figure 3**). Furthermore, the volume density of the type 2 fibers from trained is equal to (Howald et al., 1985) or higher (Nielsen et al., 2010a) than in type 1 fibers from untrained individuals. Thus, fiber type mitochondrial content is extremely malleable with muscle activity and inactivity (Hoppeler, 1986; Nielsen et al., 2010b). These changes in fiber metabolic characteristics are clearly not fiber-type-dependent, and a considerable variation exists within each fiber type with a clear overlay between fiber types. In line with this, a recent study indicated that type 2a fibers can possess equally high or even higher mitochondrial respiration as type 1 fibers (Boushel et al., 2014). The equal volume density of mitochondria and CS activity in different types of fibers suggest that the intrinsic characteristics of mitochondria are variable and not determined solely by fiber type.

Here we report that the metabolic profile of muscle fibers varies with no change in the myosin isoform they express. Thus, in highly trained humans, the mitochondrial volume percentage is equal in the arms and legs, despite a relatively higher number of MHC-2 fibers in arms, and type 2A fibers from the arm being larger, with the same number of capillaries per fiber area. In these highly trained skiers, the type 2 fibers have an equally high oxidative capacity as type 1 fibers, demonstrating that the metabolic profile of a given fiber isoform displays considerable plasticity. Interestingly, we have previously reported in the same subjects, an approximate 4.6-fold higher SR Ca^2+^ release rate in MHC 2 fibers compared to MHC 1 (Ørtenblad et al., 2011). As SR Ca^2+^ handling is a key component in the development of fatigue during most types of exercise, it is physiologically crucial for these skiers to possess a high SR Ca^2+^ uptake and release rate (Ørtenblad et al., 2000a; Gejl et al., 2014). These data on trained skiers suggest a new perspective on fiber types, indicating a divergence between MHC isoform pattern and aerobic metabolic capacity, with a high variability in the metabolic profile, closely related to the usage of the muscle fiber, within the various MHC isoforms. Thus, these highly trained skiers possess a type 2 fiber which is highly oxidative, has an equal CS activity as type 1 fibers, has a larger CSA, with the same capillarization per CSA, while having a near fivefold higher SR Ca^2+^ handling capacity than type 1 fibers. In all, these findings represent a muscle fiber with high force and power properties, while having a highly developed endurance capacity to fulfill the demands of today’s elite cross-country skier requiring the combined ability to generate and sustain rapid, prolonged high force production during short contacts with the ground (Holmberg, 2015; Andersson et al., 2016).

### Mitochondrial Subcellular Distribution and Volume Fraction

The current data from arm and leg muscles drawn from the elite endurance-trained subjects revealed that type 1 and 2 fibers have the same relative subcellular distribution of mitochondria. Thus, around 85% of the muscle mitochondria are located in the IMF region and the remainder in the SS region, regardless of fiber type and limb. This is in line with a training study showing that type 1 and 2 fibers have similar relative distribution of mitochondria after training (Howald et al., 1985).

The mitochondrial volume fraction was not different between limbs, averaging 9.5%. The reported mitochondrial volume fraction is ∼20–30% higher than found in previously reported short-term training studies (Howald et al., 1985; Nielsen et al., 2010a) as well as in endurance-trained athletes (Hoppeler, 1986). However, a mitochondrial volume percentage of 11.4% in *vastus lateralis* for a similar group of highly trained athletes, i.e., professional cyclists (*n* = 3), has been reported (Hoppeler, 1986). In these athletes, *vastus lateralis* played a more primary role in performance than in cross-country skiing, explaining the greater necessity for mitochondria in that particular muscle. The mitochondrial volume fraction of 9.5% in trained skiers is about two times larger than previously reported in untrained individuals using the same method (Nielsen et al., 2010a), and is in line with data showing a two to two-and-a-half fold higher activity of key mitochondrial enzyme (SDH, CS, and HAD) activity in trained cross-country skiers than observed in sedentary individuals (Gollnick et al., 1972; Saltin, 1996). In addition to mitochondrial distribution and volume percentage of the cell and mitochondrial enzymes, there may be other differences in mitochondria network, shape, topology, or function between fiber types, limbs, and human populations (Nielsen et al., 2017).

### Mitochondrial Content and Distribution in Leg Versus Arm

Weighing the different fiber type distribution in leg and arm muscle, the mitochondrial volume fraction was equal in both (**Figure 3D**). This suggests that arm muscles, despite lower fat oxidation capacity (Helge, 2010), HAD activity (present data), lower IMCL content (Koh et al., 2017), and higher lactate release during exercise (Van Hall et al., 2003), still require a high mitochondrial oxidative capacity. Indeed, there was a tendency (*P* = 0.095) toward a 10% higher mitochondrial volume fraction in the fibers from the arms compared with the legs (**Figure 3C**), predominantly due to a tendency to higher volume fraction in type 2 fibers in the arms (**Figure 3C**). Thus, differences in leg and arm whole-muscle metabolic characteristics may not solely be explained by the dissimilar fiber type distribution in the limbs. The high mitochondrial content in type 2 fibers in arm could either be a consequence of the high metabolic demand in the upper body of these trained subjects or, possibly, due to a high demand for glycolytic flux in type 2 fibers. Thus, there is a clear necessity for being able to convert lactate to pyruvate within the mitochondrial intermembrane space with pyruvate subsequently taken into the mitochondrial matrix where it enters the TCA cycle and is ultimately oxidized (Brooks et al., 1999; Hashimoto et al., 2006; Jacobs et al., 2013). Furthermore, peak arm blood flow and O_2_ delivery per unit muscle mass during arm exercise is higher than that to leg muscle during leg cycling reflecting the proportional matching of oxygen delivery to oxidative capacity (Boushel et al., 2011).

The present study design involved a pair-wise comparison of equally highly trained muscles from the same individual subjects, who had trained systematically for 11 years on average and whose muscle mitochondrial volume fractions are among the highest ever reported. A cross-sectional comparison of, e.g., kayakers or cyclists who train their upper or lower bodies specifically, would have allowed characterization of more highly trained muscles, for instance, with more extensive local blood flow during exercise. However, higher mitochondrial volume fractions have not been reported in larger groups and a cross-sectional design would limit direct comparisons between limbs. At the same time, it is important to note that our present observations and conclusions are relevant only for equally well-trained arm and leg muscles.

### Intramyocellular Lipid (IMCL) Content and Subcellular Localization

We have recently reported in a companion paper (Koh et al., 2017) that, in these subjects with highly trained upper and lower body, the IMCL volume fraction was fourfold higher in leg muscle than in the arm muscle. The higher content of IMCL content was apparent in both the IMF and the SS regions. Additionally, there was a fiber type specific difference in IMCL volume fractions, with a threefold higher IMF (*P* = 0.0002) and total (*P* = 0.0003) lipid droplet volume fractions in type 1 fibers than in type 2 fibers, while no difference was found between the fiber types (*P* = 0.6) in the SS lipid droplet volume fraction. The fourfold lower IMCL content of the arms compared to the leg cannot solely be explained by the higher proportion of MHC-2 fibers of arm, so a true intrinsic limb difference in fat metabolism must exist. The higher IMCL content of the leg muscle compared with the arm muscle is in accordance with the lower fat oxidation capacity of arm muscle (Helge, 2010) and the notion that exercising arm muscle evidently has a lower fat oxidation compared to leg muscle (Calbet et al., 2005; Helge, 2010).

The high content of IMCL in skeletal muscle of trained subjects and obese type 2 diabetics has been described as “the athlete paradox” (Goodpaster et al., 2001). However, the current data are in accordance with a new perspective on this apparent paradox, suggesting that the elevated IMCL content found in both type 2 diabetic patients (Nielsen et al., 2010a) and endurance-trained athletes (Koh et al., 2017) is an average of differential subcellular distribution of IMCL, where athletes have elevated IMF and type 2 diabetes patients elevated SS IMCL (Nielsen et al., 2010a). Thus, the roles of IMF and SS IMCL in skeletal muscle glucose regulation are most likely fiber type and training status specific.

### Enzyme Activities

In our highly trained cross-country skiers, with equally and exceptionally well-trained leg and arm muscle, there was a 52% higher HAD capacity in the leg compared to arm muscles and the ratio between the HAD and CS activity was 1.22 in the leg and 0.86 in arm, suggesting a relatively higher capacity for lipid oxidation in leg muscle. These enzyme activity data demonstrate a very good agreement with the four-fold higher IMCL in the leg compared to the arm muscle. Notably, the MHC-1 content was a very strong predictor of HAD activity in trained arm (*r*^2^ = 0.69), with no association in leg muscle. This clear limb difference in the association between fiber MHC distribution and HAD capacity was not apparent with CS activity, depending rather on other factors as, i.e., training-induced adaptations. Taken together, there is a clear limb difference in HAD and HAD/CS ratio, with legs having a non-MHC dependent capacity to oxidize fat, while in arm muscle, there is a very close association between MHC distribution and both absolute (HAD activity) and relative capacity to oxidize fat (HAD/CS). This supports the notion that the upper body has a lower capacity to oxidize fat and a lower fat oxidation during the same relative intensity compared to leg muscle (Calbet et al., 2005; Helge, 2010). This lower capacity to oxidize fat and higher reliance on CHO oxidation in the upper body compared to the lower body is irrespective of limb training status, and persistent also in highly trained cross country skiers with equally trained arm and leg muscle.

## Conclusion and Perspectives

Here, we show that in highly trained muscles of elite cross-country skiers, the mitochondrial volume percentage as well as the number of capillaries per fiber area are the same in the arms and legs, despite the presence of relatively more MHC-2 fibers and larger type 2A fibers in the arms. Thus, the metabolic profile of muscle fibers can vary without any change in the myosin isoform they express. These findings provide a new perspective, with a divergence between fiber type and aerobic metabolic capacity, and considerable variability in the metabolic profile of the various MHC-isoforms which is closely related to the usage of the muscle fiber. Our well-trained cross-country skiers have developed highly oxidative type 2 muscle fibers capable of producing great force and power in order to meet today’s need for pronounced endurance in combination with rapid generation of large forces during short contact periods.

We also demonstrate that leg and arm muscles exhibit a clear difference in their IMCL content and distribution, as well as in the ability to oxidize fatty acids. The observed difference in IMCL content in the upper and lower body cannot be explained by training status of the involved muscles or the different fiber type distribution in the limbs. This implies that the capacity to oxidize and store IMCL is clearly higher in leg compared with arm muscle, even though limbs are equally highly trained and express similar mitochondrial content and capillarization. In line with this, the HAD activity and the HAD/CS ratio were significantly higher in leg muscle. Thus, it is evident that limbs have different lipid metabolism independent of fiber type differences.

## Author Contributions

NØ, JN, H-CH, and BS were involved in the study design. NØ, JN, H-CH, KS, and BS have collected the data. All authors contributed to interpretation of data and drafting of the manuscript and all but BS have reviewed the final version of the submitted manuscript. BS passed away before the final approval of the manuscript. Transmission electron microscopy measurements were performed by JN at the Department of Pathology, Odense University Hospital, Denmark.

## Conflict of Interest Statement

The authors declare that the research was conducted in the absence of any commercial or financial relationships that could be construed as a potential conflict of interest.
